# Cognition Deficits in Parkinson's Disease: Mechanisms and Treatment

**DOI:** 10.1155/2020/2076942

**Published:** 2020-03-24

**Authors:** Congcong Fang, Longqin Lv, Shanping Mao, Huimin Dong, Baohui Liu

**Affiliations:** ^1^Department of Pediatrics, Renmin Hospital of Wuhan University, Wuhan, Hubei 430060, China; ^2^Department of Neurology, Renmin Hospital of Wuhan University, Wuhan, Hubei 430060, China; ^3^Department of Neurosurgery, Renmin Hospital of Wuhan University, Wuhan, Hubei 430060, China

## Abstract

Parkinson's disease (PD) is the second most common progressive neurodegenerative disorder mainly in middle-elderly population, which represents diverse nonmotor symptoms (NMS) besides such well-documented motor symptoms as bradykinesia, resting tremor, rigidity, and postural instability. With the advancement of aging trend worldwide, the global prevalence of PD is mounting up year after year. Nowadays, accumulating lines of studies have given a comprehensive and thorough coverage of motor symptoms in PD. Yet much less attention as compared has been paid to the nonmotor symptoms of PD, such as cognition deficits. Of note, a patient with PD who suffers from cognitive impairment may harbour a statistically significantly higher risk of progressing toward dementia, which negatively affects their life expectancy and daily functioning and overall lowers the global quality of life. Furthermore, it is a widely held view that cognitive dysfunction does not just occur in the late stage of PD. On the basis of numerous studies, mild cognitive impairment (MCI) is a harbinger of dementia in PD, which is observed as an intermediate state with considerable variability; some patients remain stable and some even revert to normal cognition. Considered that the timing, profile, and rate of cognitive impairment vary greatly among PD individuals, it is extremely urgent for researchers and clinicians alike to identify and predict future cognitive decline in this population. Simultaneously, early screening and canonical management of PD with cognitive deficits are very imperative to postpone the disease progression and improve the prognosis of patients. In our review, we focus on a description of cognitive decline in PD, expound emphatically the pathological mechanisms underlying cognition deficits in PD, then give a comprehensive overview of specific therapeutic strategies, and finally dissect what fresh insights may bring new exciting prospect for the subfield.

## 1. Introduction

**Figure 1:**
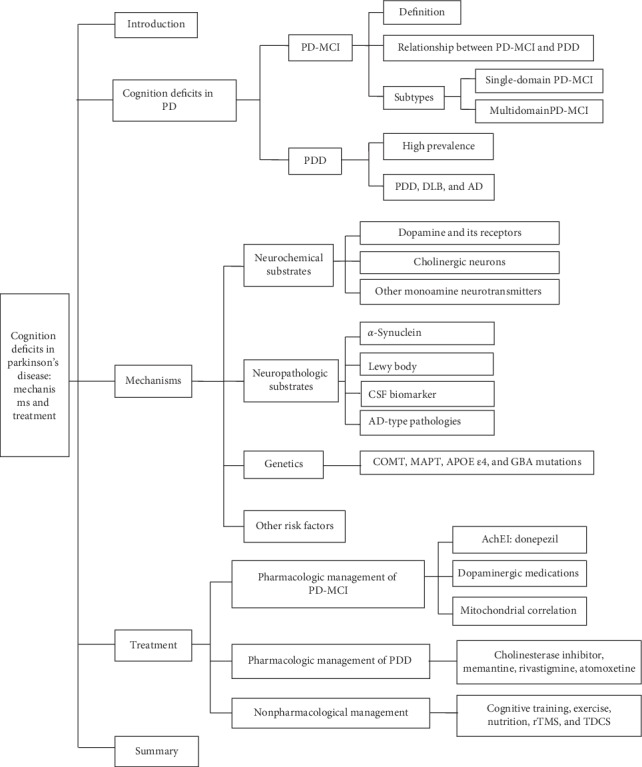
Overview of this article. PD: Parkinson's disease, PD-MCI: mild cognitive impairment in PD, PDD: Parkinson's disease dementia, DLB: dementia with Lewy bodies, AD: Alzheimer's disease, CSF: cerebrospinal fluid, COMT: catechol-O-methyl transferase, MAPT: apolipoprotein E, GBA: glucocerebrosidase, AChEI: acetylcholinesterase inhibitor, rTMS: repetitive transcranial magnetic stimulation, and TDCS: transcranial direct current stimulation.

Parkinson's disease (PD) ranks second in the common progressive neurodegenerative disorders, only after Alzheimer disease (AD), with an estimated prevalence of over 10,000,000 cases on a global scale. It is initially hallmarked by motor symptomatology which encompass bradykinesia, resting tremor, rigidity, and postural and gait disturbances (Figure 1). However, yet, currently, converging studies on the nonmotor deficits involving cognitive impairment, autonomic dysfunction, and psychiatric symptoms are launched within the field of neuroscience [1]. Cognitive dysfunction is proposed as the frequent and devastating one of nonmotor symptoms in PD. It can not only reduce the daily function of patients with Parkinson's disease but also affect family members' well-being, while it has been traditionally thought that cognitive dysfunction does not emerge until late in the progression of PD, which is well supported by the finding that more than 80% of PD individuals do evolve into dementia but only in later stages [2]. Instead, mild-moderate cognitive impairment tends to be present in early stage of the disease, which accounts for around 40% of overall PD patients [3]. In addition, recent advances in our understanding of mild cognitive impairment in PD (PD-MCI), its variable clinical presentations, and differences in progression to dementia, however, suggest that PD-MCI may not be a single, uniform entity. What fresh theory might explain this?

As we know, the etiopathogenesis of PD characterized by irreversible disease progression is quite complicated and still lack of consensus to date, especially on the cognitive deficits of PD. A growing chorus of up-and-coming scholars ascribes the pathogenesis of PD to multineuropeptide dysfunction. Put another way, it is not only the progressive deterioration of dopaminergic neurons but defects in nondopaminergic systems that can lead to classical motor and nonmotor manifestations [1]. Robust research component demonstrates that the early reduction of dopaminergic uptake in the frontal lobes is of crucial importance to cognitive impairment existing in PD patients [4]. Accumulating lines of evidence suggest that the cholinergic disturbance within brainstem and corticostriatal pathways may be implicated in the pathophysiology of cognition deficits in PD [5, 6]. In addition, recent progress in PD has revealed that several genetic biomarkers and gene polymorphism may be connected with the generation and development of PD with cognition deficits.

All in all, since the underlying pathogenesis of cognition decline is largely ambiguous, adding that the treatment options targeting cognitive dysfunction are quite limited, the neurodegenerative disease has brought unduly socioeconomic burdens and immeasurable family suffering. In conjunction with the present grim situation, it is essential and urgent for more pundits to further explore and summarize the possible mechanisms which underlie the onset and evolution of the cognitive dysfunction in PD for establishing more standardized management tactics.

## 2. Cognition Deficits in Parkinson's Disease

Cognitive dysfunction is one of the major clinical nonmotor symptoms of PD with insidious onset, including mild cognitive impairment and dementia, especially for executive dysfunction with coexisting other cognitive domains impairment such as speech dysfunction, visual spatial ability, and memory impairment. Cognitive dysfunction is increasingly found prevalent in PD individuals and even in clinical newly diagnosed PD patients [7]. Emerging epidemiologic study indicates that the cumulative prevalence of Parkinson's disease dementia (PDD) in 8 years is as high as 78.2% [8]. Roughly 40% of PD subjects at an earlier stage have co-existing mild cognitive impairment, boosting the risk of converting to PDD [9]. Cognitive deficits in PD has certain heterogeneity as described in Kehagia's proposed dual syndrome hypothesis [10]. PD with cognitive impairment can be divided into two broad categories: one is given priority to planning, working memory, and executive dysfunction that is related to the frontal lobe-striatum loop coupled with decreased dopamine levels and the other is disturbances of attention, semantic verbal fluency, and visual spatial ability with involvement of not only the temporal lobe but the posterior cortical dysfunction.

### 2.1. PD-MCI

Although cognitive dysfunction in PD has been widely noted for a long time, PD-MCI only emerges more recently as a distinct entity with a mean prevalence of 27% [11]. PD patients who are diagnosed with PD-MCI exhibit subtle cognitive decline compared with the premorbid state, but do not accompany comorbidities impacting on cognitive function and not have significantly functional deficits enough to meet diagnostic criteria for PDD. PD-MCI is defined classically as a prodromal or transitional state, which is a common occurrence in early or newly diagnosed PD patients with slightly impaired cognition but can increase the risk for developing subsequent dementia. The diagnostic criteria for PD-MCI in numerous studies were a 1.5 standard deviation or greater deficit upon neuropsychological testing consistently across at least one cognitive domain without dementia [12].

As a matter of fact, PD-MCI is also a nonmotor complication of PD but newly proposed as a potential harbinger of conversion to PDD. PD- MCI displays a certain degree of cognitive decline that does not run in parallel with the extension of aging in subsets of participants, whereas not all MCI are destined to progress to dementia [13]. It is reported that up to 80% of PD-MCI patients can convert into PDD [11]. Therefore, early identification of PD-MCI patients who may be converted to PDD and targeted treatment is particularly important. The International Parkinson and Movement Disorder Society defined specific guidelines for PD-MCI as level I (abbreviated assessment) and level II (comprehensive assessment) categories [11]. Some studies have found that level II criteria can be used as a prognostic indicator of cognitive decline in PDD, which is affected by age, gender, years of education, depression, and severity of PD [14]. Males with Parkinson's disease are more likely to develop dementia. Dopamine depletion in PD impairs frontal functions such as executive function and attention by affecting the frontostriatal pathway. Therefore, damaged processing speed and working memory is associated with PD-MCI conversion to PDD [15]. Other researchers have found that decreased semantic fluency, impaired drawing, memory, and visuospatial function in PD-MCI patients are in connection with the occurrence of PDD [16]. Chung and his colleagues used magnetic resonance imaging (MRI) to compare patients who had converted to PDD with PD-MCI patients and found that the frontal cortex was thinner [17]. Taken together, these findings suggest that structural impairment of the frontal, temporal, or parietal lobes can lead to the transition from MCI to dementia in PD patients. Moreover, *α*-synuclein levels can also be used as a biomarker to predict cognitive impairment in PD patients [18]. Remarkably, lots of latest research work reveal that PD-MCI may represent a variable course for PD patients, a rather static condition in someone, a short-term improvement in others, or conversion to dementia for many of them [19, 20]. Identifying the early stages of MCI may provide the possibility for early intervention to improve cognitive reserve, protect cognitive status, or prevent cognitive decline.

According to the different failure in cognition domains, PD-MCI can present the following two subtypes: (i) single-domain PD-MCI, namely, the single cognitive domain with two abnormal test results, where other cognitive domains were not damaged; and (ii) multidomain PD-MCI—two or more have at least one test abnormity. Of note, the clinical manifestations of PD-MCI phenotype are heterogeneous with defects in multiple cognitive domains, and some exhibit obvious amnestic deficits. PD-MCI distinguishes from AD with defects in subfrontal cortex region for attention and execution functioning, which may be accompanied by visuospatial ability and memory impairment [21]. There exists a single or multiple cognitive domains in PD patients with MCI, of which executive dysfunction and memory impairment stand out with a similar incidence. However, several studies have shown that executive dysfunction occurs more frequently than memory disorders [22, 23].

With the frequency and clinical phenotypes of PD-MCI having been explored in extensive studies of the past, factors influencing the heterogeneity of PD‐MCI include genetics, premorbid functioning/cognitive reserve, the environment, and definitions used for PD-MCI [24], while the thorough pathogenetic mechanisms are not well investigated. Currently, research studies increasingly suggest that the imbalance of neurotransmitters may largely contribute to the occurrence and progression of MCI in PD patients, but which is deserved to be verified [25]. In response to these claims, existing pharmacological and nonpharmacological measures have been identified to be typically marginal and nonsustained coupled with potential adverse effects.

In combination with the current situation, it is essential and urgent for more pundits to further explore and summarize the possible mechanisms underlying the onset and evolution of PD-MCI in order to establish more standardized management tactics.

### 2.2. PDD

PDD exhibits a more devastating cognitive impairment, which has been identified as a late manifestation of PD with high prevalence, disability rate, and poor response to treatment. A subset of patients who are diagnosed with PD or PD-MCI can eventually deteriorate into PDD along with the extension of disease course. The epidemiological survey conducted by Aarsland et al. [26] showed that PD patients suffered from dementia with a high prevalence ranging from 24% to 31% for rough estimation. In another prospective multicentral clinical study from Sydney, Australia, 136 cases of PD patients with definite pathological diagnosis were recruited, approximately 28% of whom inevitably progressed to dementia after a follow-up of 5 years, nearly 48% after 15 years of follow-up, and over 83% after 20 years of follow-up [2]. But interestingly, Reid et al. [27] reported that patients with PD would convert to dementia at the age of 70 or so, implying that dementia had nothing to do with the time of onset of PD. PDD is one relatively common nonmotor feature for PD individuals with a high frequency in later disease stages, which further increases the physical and psychological stress on patients and their families. There is stark evidence that PD patients with dementia can still double the risk of mortality in PD counterparts who do not develop dementia after adjusting for the motor symptoms [28]. Although the two patterns of cognitive impairment of early (5–10 years after the occurrence of PD) and late (after 10 years) onset of PDD are similar, the former is more serious than the latter in speech dysfunction before coexisting with dementia [27].

Of note, PDD has always been confused with dementia with Lewy bodies (DLB) both in the clinical practice and clinical studies (Table 1). It is usually identified by the priority of the occurrence of dementia in diseases, namely, the diagnosis of PDD is established if dementia appears after 1 year in the context of PD, otherwise is diagnosed with DLB. Other than the severity of the disease, PDD behaves various informatively with multiple cognitive domains disturbance similar to AD-not only executive dysfunction, but memory, attention and visuospatial disorders. One recently published study comparing the neuropsychological profile of PDD and AD reported that the deterioration of executive function and visuospatial skills is more present in PDD, but there were no significant difference in memory impairment (Table 1).

As a matter of fact, the pathologic mechanism of PDD is complex as well as various, and there remain some limitations to strong evidence for the clinical schedules.

## 3. Mechanisms

To date, it is still an ongoing debate as to the pathogenesis of cognitive dysfunction in PD. Strikingly, with the deepening of relevant studies of late years, numerous scholastics believe that not only neurochemical alterations in dopaminergic, cholinergic, and other systems but also neuropathological contributions of limbic and cortical Lewy bodies and neurites, amyloid deposition, neurofibrillary tangles, and even cerebrovascular disease, mitochondrial dysfunction, inflammation, and neurotrophic factors have frequently given rise to cognitive deficits in PD.

### 3.1. Neurochemical Substrates

#### 3.1.1. Dopamine and Its Receptors

**Figure 2:**
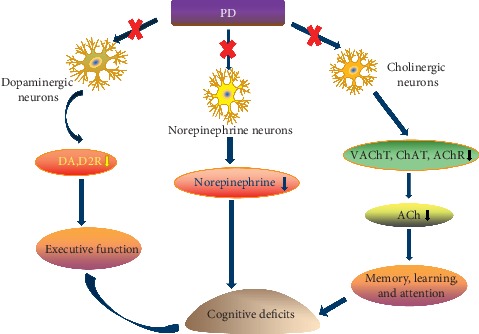
The main neurochemical substrates of cognitive dysfunction in PD. PD: Parkinson's disease, DA: dopamine, D2R: dopamine2 receptor, ACh: acetylcholine, VAChT: vesicular ACh transporter, ChAT: choline acetyltransferase, and AChR: acetylcholine receptor.

Extensive literature of the previous years has emphatically discussed the relationships among executive function and dopamine. One study undertaken recently has found that the denervation of striatal dopamine and D2 receptor deficiency in insula lobe region may strongly correlate with cognitive decline of PD-MCI (Figure 2), particularly affecting executive functioning [25]. A research with functional magnetic resonance imaging (fMRI) showed that the neural activity of prefrontal cortex, striatum, and thalamus in frontal⁃striatum regions decreases when executive dysfunction of PD patients is tested with task-switching and working memory test [29]. Several PD studies support that dopamine in caudate nucleus reduces or even disappears during objects conversion or the Stroop color-word test (SCWT). Hall [30] has also reported that PD patients with dopaminergic neurons deficiency in the ventral tegmental area (VTA) tend to convert to dementia at autopsies. Neuroimaging studies using PET showed that there is a reduction in metabolism of D2 receptors in insula lobe of PD with cognitive impairment, especially executive dysfunction, indicating the expression changes of dopamine in midbrain-cortex circuits are connected with cognitive dysfunction in PD [25].

Another study showed that a deficiency of nigrostriatal dopaminergic neurons was closely correlative to impaired executive performance, but had no relation to memory and visual-spatial measures in early PD patients [31]. More importantly, any state of dopaminergic insufficiency, such as preadministration of levodopa or other dopaminergics and withdrawal of dopaminergic therapies, has been proven to be a causal relation to cognitive decline in PD [32].

#### 3.1.2. Cholinergic Neurons

It is assumed that the disrupted ascending cholinergic pathway is of great importance to progress to dementia of PD, which is supported by neuroimaging findings that patients with PD and PDD both have cholinergic neuron deficits with vesicular acetylcholine transporter (VAChT) and acetylcholinesterase (AChE) [33, 34]. Moreover, PD patients without dementia only display decreased VAChT uptake in the parietal and occipital lobe, whereas there is more substantial decrease in extensive cerebral cortex of PDD individuals [35]. Hilker et al. [36] have reported nondemented PD patients are always accompanied by cortical cholinergic dysfunction,which is more significant in PD patients with dementia. 

Take Ach for example; a neurotransmitter inextricably related to memory and learning can help ensure the functional integrity of the central nervous system through stimulating cholinergic nerves and ultimately alleviate the symptoms of executive dysfunction, memory deficits, and reduced mental flexibility. The insufficiency of Ach receptors in such anatomic regions as the thalamus, midbrain, temporal cortex, hippocampus, and cerebellum was tightly associated with the severity of cognitive deficits in PD-MCI [37]. Besides, the whole cholinergic system has been closely implicated in cognition declination in PD. Not only cholinergic deficits in the basal forebrain and prefrontal cortical areas but also the decreased activity of choline acetyltransferase (Figure 2) in the frontal and temporal lobes may be responsible for PD-related attention, learning, and memory impairment [38].

#### 3.1.3. Other Monoamine Neurotransmitters

Norepinephrine (Figure 2) is an excitatory neurotransmitter mainly released by the locus coeruleus (LC), followed by projecting to the hippocampus cortex, cingulate cortex, and neocortex, which may involve in maintaining memory, attention, and normal behavior via oxidative metabolism and neuroimmune system [39]. Buddhala et al. [40] carried out a research with 15 cases of cognitively impaired PD individuals and 6 cases of normal controls enrolled for autopsy and detected a decreased level of norepinephrine in many brain regions by the methods of high-performance liquid chromatography (HPLC) and enzyme-linked immunoabsorbent assay (ELISA), indicating the deletion of norepinephrine is one of mechanisms of PD with cognitive disorder.

It is a known neuroanatomy theoretic that B6/7 neurons in the dorsal raphe nucleus project to the striatum and the cerebral cortical regions with B5/8 neurons in the raphe nuclei projecting to the cerebral cortex and hippocampus. Research has long found that B5/8 5-HT neurons in the raphe nucleus of late-stage PD patients were increasingly lost along with the evolution of PD, while B6/7 neurons did not have pathological lesion [41].

### 3.2. Neuropathologic Substrates

Previous autopsy study on PD found that the presence of *α*-synuclein in brain tissue is positively correlative with the severity of the condition [42–44]. The deposit of *α*-synuclein may affect the cerebral cortex function to an extent, leading to cognitive impairment. The nerve fiber loss of subcortical nuclei projecting into striatum, limbic system, and midbrain can also render cognitive disorder [45], which highlights the roles of neurochemistry and neuropathology in the pathogeny of PD-CI. Further research shows that *α*-synuclein is currently being used to diagnose dementia of Parkinson's disease with high sensitivity and specificity [46]. Studies have found that *β*-amyloid (A*β*) deposition in PD patients is associated with impairment of attention [47], while Melzer et al. think there is no relationship between A*β* deposition and impaired cognition [48]. There is a contradiction between the two conclusions. This may be due to different research methods, and it needs further exploration.

Lewy body related pathological changes are proposed as the most important factor in the progress of cognitive dysfunction in PD. Hurtig et al. [49] in 2000 have confirmed firstly Lewy body relative to *α*-synuclein is widespread in the cerebral cortex, especially the frontal lobe and cingulate, whose cognitive dysfunction caused by the related pathology change is unlike Alzheimer's disease. However, note that not all PD patients with Lewy's body in brain cortex detected by pathological autopsy show cognitive dysfunction. Therefore, there are still plenty of questions as to the relationship between Lewy's body related pathological variation and the cognitive dysfunction of PD.

Studies have also shown an association between cerebrospinalfluid (CSF) biomarker and progression of cognitive impairment in PD, which mainly focus on A*β* 42, *t*-tau, *p*-tau, *α*-synuclein, and neurofilament light chain (NFL). High CSF tau and phospho-tau were concerning with impaired memory and naming in PDD patients, while low CSF A*β* was related with phonetic fluency in nondemented patients [50]. Low baseline CSF A*β*42 is predictive of cognitive decline in most nondemented PD patients [51]. A research indicated higher CSF *p*-tau and *p*-tau/A*β*42 could predict subsequent reduced memory and executive function among patients who had just started levodopa therapy [52], while Parnetti et al. discovered no association between CSF levels of tau and subsequent cognitive decline, which may be due to the absence of more sensitive methods [53]. Future studies will hopefully combine CSF data with pathology to increase our understanding of the molecular mechanisms of PDD.

In addition to cerebrospinal fluid, researchers found high levels of plasma A*β*42 and total tau could also predict cognitive decline in amnestic MCI [54]. A cross-sectional study utilising immunomagnetic reduction (IMR) method for the determination of plasma *α*-synuclein found that *α*-synuclein levels were negatively correlated with cognitive function. Therefore, plasma *α*-synuclein levels can also be used as a noninvasive biomarker to predict cognitive impairment in PD patients [18]. Additionally, the pathological changes highly associated with Alzheimer's disease (AD) display a crucial role in the cognitive dysfunction of PD patients, which can be accompanied by Lewy's body in the same context [55–57]. Compta et al. [56] have detected Lewy body coexists with A*β*, *α*-synuclein and tau-protein in 56 cases PD patients (29 cases with PDD) well diagnosed by pathological examination, all of which are the most relevant pathologic features associated with PDD confirmed by the following Cox regression analysis. According to a meta-analysis from the UK, a mix of AD-related pathology change and Lewy's body is the most solemn part of neurodegeneration, and A*β* deposition in cerebral cortex can further accelerate the progression to PDD [57]. It is suggested that AD-type pathologies may play a specific role in the cognitive dysfunction of PD cohorts greatly affected by co-morbid Lewy body.

### 3.3. Genetics

It has been well documented that genetic polymorphisms correlative with dopamine regulation and tau proteins contribute to the occurrence and development of the cognitive dysfunction in PD. The COMT genetic polymorphisms with different variants may have divergent impact on executive function tasks by modulating the activity of the dopamine-related enzymes in the prefrontal cortical areas. Val/Val polymorphisms may boost dopamine catabolism with consequent loss of postsynaptic dopaminergic stimulation, whereas Met/Met polymorphisms lower the activity of COMT enzyme with increased dopamine levels [58]. Given the above, the COMT genotype is not necessarily associated with subsequent cognitive impairment and even dementia with a follow-up of 5 year in the CamPaIGN cohort.

However, tau-related MAPT gene polymorphisms always coexist with greater posterior cortical cognitive deficits. It has been reported MAPT H1/H1 genotype shows stronger associations with PD dementia in the same cohort [59, 60]. Additionally, MAPT H1 homozygotes have been found to exhibit a direct causal relationship with the decreased activity of the posterior visual network during visuospatial tasks [61].

Another study recently demonstrates that there is a significant reduction in functional activity of the medial temporal lobe (MTL) network in those newly diagnosed PD patients with the APOE *ε*4 allele when undergoing certain memory tasks. Nevertheless, plenty of pundits say the finding is a highly controversial one which merits replication in larger studies [61]. Clinical and pathological evidence also suggests that diffuse neocortical Lewy body-type pathology tends to occur more frequently in patients with Parkinson's disease with GBA mutations. It has been linked to hallucinations, cognitive decline, or dementia in PD patients [62]. Mata et al. found GBA variants are associated with cognitive impairment in working memory, executive function, and visuospatial abilities, which verifies GBA-related cognitive impairment [63].

Most of these genetic biomarker findings do reveal an intimate association of genetic alterations and cognitive decline in PD, which can be used to predict disease development and evaluate the severity. Taken together, we have had a basic knowledge of PD-MCI and PDD related mechanisms explaining their pathogenesis and disease progression, which carries a profound implication for seeking new avenues for earlier intervention and more individualized treatment. MAPT H1/H1 genotype is proposed as an independent predictor of dementia, which exerts effects on tau transcription functionally. By contrast, COMT genotype has a significant impact on an executive task based on frontostriatal system but with no effect on dementia. These research efforts have proved conclusively that the dementing process of PD is highly predictable through the above informative predictors with the hope of translating into the clinic one day [59].

### 3.4. Other Risk Factors

Older PD patients especially with longer disease duration are more prone to suffer from dementia. Compared with men, it takes a relatively extended period of time for women to develop dementia. Smoking, alcohol consumption, cardiovascular disease, and cerebrovascular disease may synergistically relate to cognitive impairment in PD patients [64]. Higher Hoehn–Yahr grading and lower cognitive functioning scores can add the risk of dementia. In addition, the variants of motor symptoms have marked association with the onset of PDD, of which masklike face, bradykinesia, or akinesia would be more easily complicated with dementia. Not only that, but the patient's mental state, drug susceptibility, and adverse effects may also exert a crucial role in the occurrence of PDD. PD patients with poor response to dopamine agonists are more likely to develop PDD [65, 66], and many adverse reactions of dopamine or other drugs such as delirium and visual hallucination are also correlated with the occurrence of PDD [67]. It was found that PD patients with depressive state of different extents were more inclined to develop dementia [68].

## 4. Treatment

Various treatments developed for ameliorating the motor symptomatology of PD patients has been introduced for over 40 years, all of which are proven to be effective. However, reasonable standard treatments for many “nonmotor” deficits still remain highly indefinable. Furthermore, established research studies have claimed nonmotor clinical symptoms respond poorly to aforementioned approaches and probably can be exacerbated [60].

Many of the pharmaceutical therapies developed for PD-MCI and PDD, cholinesterase inhibitors and memantine included, have been tested in MCI and AD patients. Currently, such therapeutics as rasagiline, rivastigmine, droxidopa, and PDE4 inhibitors have been tested for PD-MCI. To date, Food and Drug Administration (FDA) states that rivastigmine is the only approved but modestly effective agent for PDD. In addition to pharmacological treatments, the extensive research into nonpharmacological interventions such as cognitive training, exercise programs, and brain stimulation techniques is ongoing [69–71].

### 4.1. Pharmacologic Management of PD-MCI

To date, it is surprisingly slow in the advancement of therapeutic interventions specifically developed for PD-MCI. Not only that, but existing pharmacological and nonpharmacological measures have been identified to be typically marginal and nonsustained coupled with potential adverse effects.

As mentioned earlier, the pathogenesis of PD-MCI may involve the changes of cholinergic neurotransmitter, which may provide a theoretical basis for its pharmaceutical therapy. There is ample evidence that the disturbed cholinergic system may be the important cause of cognitive decline in patients with PD. Donepezil is the second acetyl cholinesterase inhibitor (AchEI) developed for improving cognitive function of PD patients, which has been approved by the FDA. It can selectively inhibit the degradation of acetylcholine in the central nervous system and effectively increase the concentration of acetylcholine in the synaptic cleft of nerve cells and delay progressive cognition impairment of PD patients with less adverse reaction and more obvious clinical safety and good tolerance. One clinical research trial has reported that cholinesterase inhibitors can benefit PD patients with cognitive impairment by significantly slowing the loss of Mini-Mental State Examination (MMSE) score, but the treatment with memantine does not work, both of which can improve the overall clinical symptoms of PD patients [72].

However, it is indisputable that the therapeutic effects of dopaminergics highly vary in cognitive impairment in PD patients. Some believe that dopaminergic medications may improve, in varying degrees, working memory, planning tasks, and cognitive flexibility in PD patients [73]. Another caveat does carry some weigh that treatment with dopamine may worsen such nonspecific symptoms as decision making or choice reaction times or showing no significantly therapeutic effect on cognitive slowing [74, 75].

Several research reports claimed that cognitive impairment in Parkinson's disease is associated with impaired mitochondrial function. As an important role in improving mitochondrial energy metabolism and decreasing oxidative stress, creatine and CoQ10 may be a new neuroprotective agent to delay PD-MCI progression [76]. It still needs a richer sample size and further studies to confirm.

In general, there is currently no specific therapeutic agent for PD-MCI. Studies that have been conducted vary in terms of interventions, participant characteristics, and outcome indicators. In the future, larger sample study and more extensive research programs are needed to improve this field. At present, identifying which groups of PD-MCI patients are more likely to be converted to PDD is of great significance for guiding clinical treatment.

### 4.2. Pharmacologic Management of PDD

There are increasing literature studies about PDD treatment at home and abroad. It is believed that one of the main solutions is to control its motor symptoms, and the other is to delay the development of cognitive impairment.

The mainstay of therapies for PDD is drug administration, including cholinesterase inhibitor and memantine. Patients with PDD have more decreased cholinergic levels of the cerebral cortex than AD individuals, which may serve as the biological basis of the effectiveness of anticholinesterase drugs [77].

Rivastigmine, the second generation of central acetylcholinesterase inhibitor, selectively inhibits cholinesterase in the cerebral cortex and hippocampus. A large randomized controlled trial (RCT) showed that rivastigmine can significantly improve overall cognitive function and clinical manifestations of PDD compared with placebo, which is the only medication confirmed to be effective for PDD [78]. Atomoxetine can selectively inhibit the reuptake of norepinephrine, a neurotransmitter related to cognitive function. There was also a systematic review which described atomoxetine had positive effects on executive function in multiple RCTs [79]. Whether these therapies can slow the progression of transition from PD-MCI to PDD is a key question for future research.

### 4.3. Nonpharmacological Management

Aside from pharmacological management, there is increasing interest and awareness of nonpharmacological interventions for cognitive impairments in PD. Nonpharmacological therapies encompass noninvasive therapies like cognitive rehabilitation, physical rehabilitation, exercise, and brain stimulation techniques and invasive treatments, such as surgery and deep brain stimulation. Nowadays, there is systematical evidence that nonpharmacological interventions are already approved to treat AD and other dementias, but the research efforts of their efficacy for PD with cognitive decline are ongoing.

Cognitive training studies based on computer and “pen and pencil” indicated that it improved executive function, working memory, and processing speed in Parkinson's patients and even reduced the risk of developing MCI [80]. However, it should be noted that extensive and randomized controlled research studies are still lacking.

A growing body of evidence has showed that proper exercise can not only improve the motor symptoms of PD patients but also ameliorate cognitive function, especially the executive function. Specifically, exercise programs have been increasingly shown to be a feasible treatment option to improve overall well-being and potentiate cognitive functions in clinical PD populations, while the underlying neuroprotective mechanisms are unclear [81, 82]. Such exercise interventions as aerobic, resistance, and dance can substantially promote neuronal proliferation and neurogenesis, which may be highly recommended as an essential part of routine management and neurorehabilitation of those PD patients with cognitive dysfunction [83].

Cognitive decline in PD is often accompanied by changes in brain structure. In brief, it is divided into changes in gray matter, which mainly occurs in the temporal region including the hippocampus, frontal lobe, and parietal lobe, and white matter, which mainly focuses on the corpus callosum and cingulate gyrus [84]. Exergames, a security technology that combines cognitive training and physical exercise, has been applied to PD's recovery exercise in recent years. It has been reported that exercise intensity is associated with an increase in gray matter volume in prefrontal cortex and anterior cingulate cortex, and improvement in speech memory is in connection with an increase in dorsolateral prefrontal cortex [85]. Several RCTs have shown that exergames can improve the cognitive function of PD patients [86, 87]. Compared with the training using a computer program, patients who used a computer sports game performed better in attention, but there was no significant difference in working memory and executive function. Another study took advantage of a 6-week home-based training period of exergaming, which found that it can improve deficits in cognitive-motor dual-tasking and attention in PD [88].

Several studies in recent years have demonstrated a link between nutrition and cognition. Some researchers have linked consumption of aquatic products such as fish to a reduced risk of cognitive impairment [89]. A meta-analysis concluded that hyperhomocysteinemia was related to cognitive impairment of PD patients [90]. Vitamin D, folic acid, and blood uric acid were also taken into account. PD cognitive dysfunction and the relationship between specific dietary patterns still need further research.

Taken together, nonpharmacological therapies, including cognitive training, exercise programs, exergaming, nutrition, repetitive transcranial magnetic stimulation (rTMS), and transcranial direct current stimulation (TDCS), and so on, can improve cognitive function, whereas whether these interventions can be used for PD-MCI or PDD requires large-scale and high-quality clinical trials to further validate [71].

## 5. Summary

Cognitive impairment, one type of nonmotor feature, which is etiologically associated with dopaminergic and nondopaminergic systems, frequently appears in PD patients with various severity levels and specific manifestation. Initial cognitive deficits, including PD-MCI, may be improved in the short term or remain stable, but in many cases, may progress to PDD over a variable period of time. Although, in the short term, nonmotor manifestations are at least as detrimental as motor symptoms for PD patient's health and quality of life, it remains difficult to manage. Currently, there are relatively lack of existing well-defined or remarkable therapies for milder forms of cognitive impairment and PDD. But above all, large observational studies have established the foundation for boosting our understanding of the progression of cognitive disorder, the impact on incident PD patients, and the underlying neurobiological mechanisms. Moreover, incorporating findings of longitudinal follow-up studies can help identify cognitively impaired PD cohorts earlier, offer a proactive preventative management, and ultimately pursue neuroprotective and disease-modifying therapies for PD-MCI and PDD.

All in all, an established therapeutic schedule needs rigorous studies. There is still a long way for clinicians to bring more improved and uniformed management into practice. It is imperative for the neurologic authorities to illuminate the mechanisms triggering PD-MCI or PDD and develop more therapeutic interventions for future clinical trials.

## Figures and Tables

**Table 1:** Comparison of clinical features of PDD, DLB, and AD.

Disease	Clinical features
PDD	Dementia appears after 1 year in the diagnosis of PD. Apathy, depression, and anxiety appear more commonly than in DLB patients.

DLB	Fluctuating cognitive dysfunction visual, hallucinations, and Parkinson's syndrome. It progresses faster than PDD, with irritability and unstable mood.

AD	AD behaves multiple cognitive domains disturbance mainly including memory, visual spatial ability, language, and executive function. The impairment of visual spatial ability and executive function was less than that of PD.

PDD: Parkinson's disease dementia; DLB: dementia with Lewy bodies; AD: Alzheimer's disease.
